# Reply to: “Assessment of heart rate variability and occurrence of falls in Alzheimer's disease: an exploratory study”

**DOI:** 10.1055/s-0046-1829033

**Published:** 2026-09-25

**Authors:** Evelize Antunes Rodrigues, Aline Roberta Danaga, Etiene Farah Teixeira de Carvalho, Carlos Alberto Santos Filho, José Burgos Ponce, Alessandro Ferrari Jacinto

**Affiliations:** 1 Universidade Estadual Paulista, Faculdade de Medicina de Botucatu, Botucatu SP, Brazil.; 2 Universidade Federal de Alfenas, Alfenas MG, Brazil.; 3 Universidade Nove de Julho, São Paulo SP, Brazil.; 4 Centro Universitário de Adamantina, Adamantina MG, Brazil.

Dear Editor,

We would like to thank Drs. ER and SM for their interest in our study titled “Assessment of heart rate variability and occurrence of falls in Alzheimer's disease: an exploratory study.” Your observations are fundamental to improve the scientific debate on heart rate variability (HRV) and the risk of falls in Alzheimer's disease (AD). Our clarifications, organized by the topics raised, are presented as follows:

Regarding the sample size and potential selection bias, we reiterate that, as highlighted in the title and objectives, we conducted an exploratory study. The final sample consisted of 26 participants obtained after the exclusion of 9 individuals due to strict technical necessity: 6 because of interference in the transmitted signals and 3 due to motor restlessness in the supine position. Although we acknowledge the absence of an a-priori sample size calculation, these exclusions were essential to ensure the methodological validity of the HRV spectral analysis, avoiding artifacts that could compromise the accuracy of the recordings obtained via portable monitor, a method that has already been validated in comparative studies 1 with the conventional electrocardiogram.

Considering the study design, we agree that the cross-sectional nature and retrospective reporting of falls over the past 3 years restrict the interpretation of causality. However, in order to mitigate recall bias (critical in AD patients), data collection was based on combined information from elderly individuals and their caregivers, as well as on the consultation of medical records. Although health status is dynamic, this investigation is justified by the high incidence of falls in this population, 2 and by the fact that the study was one of the first to analyze this specific relationship with HRV.

With respect to confounding factors, data on sedentary lifestyle and neuroleptic use were reported transparently, revealing significant differences between the groups. As acknowledged in the “Limitations” section, the small sample size prevented the application of multivariable models to adjust for these confounders. We chose to retain these participants to preserve the clinical reality of patients with AD and the polypharmacy typical of this population, an approach that is also supported by other researchers in the field, 3 who have reported the frequency of autonomic dysfunction in these patients regardless of medication use.

Regarding the statistical approach, a comprehensive analysis of various domains was intentional to identify as many potential associations as possible in this relatively-unexplored field. The application of parametric and nonparametric tests (such as analysis of variance [ANOVA], Mann-Whitney and Wilcoxon) was guided by the nature of the distribution of each specific variable. In addition, signal processing and interpretation of HRV components followed the guidelines of the Task Force of the European Society of Cardiology and the North American Society of Pacing and Electrophysiology. 4 During the exploratory phase, we prioritized minimizing the risk of type-II error to avoid omitting physiological trends that may be clinically relevant.

Finally, regarding the absence of CIs, we clarify that this choice was based on the asymmetrical nature and high dispersion of the HRV data. As detailed in our results, we used the Gamma distribution and Wald tests to analyze these interactions (Tables 4,5). Under these conditions, the CIs become excessively wide, potentially masking significant differences that *p* -values can detect with greater decisional precision for this dataset.

In summary, the study demonstrated that the use of portable monitors is a viable and promising technical alternative for AD patients. Although the findings indicate important preliminary associations, we recognize that the influence of polypharmacy 5 and other confounding factors should be validated in prospective longitudinal studies involving larger cohorts. We believe that attention to the methodological aspects discussed in this letter will contribute to a better understanding of the complex autonomic interactions in this population.
